# Twice times two: Dual mechanism for double rhythmic meter in orangutans and the evolution of human song

**DOI:** 10.1016/j.isci.2025.114273

**Published:** 2025-11-28

**Authors:** Chiara De Gregorio, Adriano R. Lameira

**Affiliations:** 1 ApeTank, Department of Psychology, University of Warwick, Coventry CV4 7AL, UK

**Keywords:** Wildlife behavior, Zoology, Anthropology

## Abstract

Rhythmic pulse, the division of a beat into subordinate patterns, is the backbone of music. Across the world’s musical traditions, the division of the primary beat into two equal parts – “double meter” – represents a prototypical pulse, also found in singing nonhuman primates. The last great ape common ancestor was, however, a non-singing species. How rhythmic pulse evolved in human song and music is, thus, enigmatic. Here, we analyze wild male orangutan long calls, which are structurally isochronous (i.e., with a steady of 1:1 rhythm). Males divided the primary rhythm into 1:2 and 2:1 subordinate patterns and did so by two distinct mechanisms: tempo changes as used by other primates and voiced in-exhale alternations as still used today by some human song traditions. Findings confirm double-meter in a non-singing great ape and suggest the two-phase cycle of the phonatory-respiratory system may have been leveraged for the evolution of human song and music.

## Highlights

• Orangutan long calls show isochrony and double-meter rhythmic patterns
• Double meter achieved via tempo shifts and inhale-exhale voicing alternation
• Findings reveal rhythmic pulse in a non-singing great ape species
• Human song rhythm may derive from ancestral phonatory-respiratory cycles

## Graphical abstract

**Figure:**
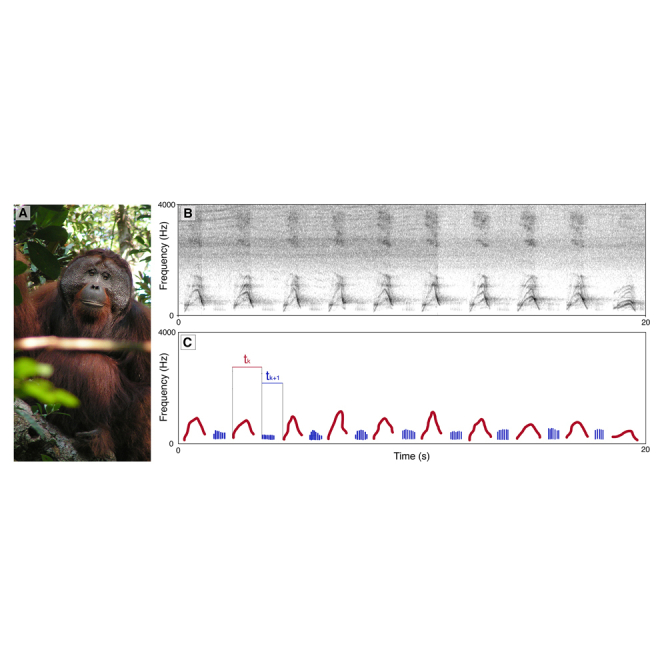

## Introduction

Music plays an important emotional and social role across human societies, shaping cultural identities. This role depends, however, on an essential feature of music – rhythmic pulse,^1^ the measured division of a beat into subordinate patterns. Rhythmic pulse provides the cadential framework that organizes sound and holds melody and harmony in place, guiding movement, entrainment, and synchronization between individuals and instruments. Across the world’s musical traditions, the division of the primary beat into two equal parts – “double meter” – represents a prototypical rhythmic pulse, presumed to be quasi-universal across the world’s music traditions.^1^^,^^2^

In nature, simple vocal rhythms based on isochrony, a steady pattern of sound units separated by equal intervals (1:1), like the ticking of a clock or a metronome, are widespread across taxa (e.g., birds,^3^ seals,^4^ bats,^5^ rodents,^6^ primates^7^^,^^8^^,^^9^^,^^10^^,^^11^). Other, more complex rhythms are, however, particularly rare. To date, double meter (1:2 and 2:1) has been identified outside human music, namely, in two species of singing (nonhuman) primates (Indri lemurs, *Indri indri*^7^ and Hainan black-crested gibbons, *Nomascus hainanus*^12^). There is no current evidence for double meter in (nonhuman) great apes, despite closer relatedness with humans. Unlike indris, gibbons, and humans, who can exhibit a double meter in their songs, great apes do not sing. Accordingly, the last great ape common ancestor is also presumed to have represented a non-singing species. How rhythmic pulse evolved in humans is, thus, equivocal. Did song emerge first than double meter in the human lineage, or did double meter precede human song? If the latter, what type of displays might have provided the precursor structures for song and music evolution? Were these displays vocal?

To answer this open question on the origins of double meter and song within the Hominid family, we conducted rhythmic analyses of the loud vocal displays of wild male orangutans – “long calls.” Orangutans are the most arboreal and solitary great ape, native to the tropical rainforests of Sumatra and Borneo in Southeast Asia,^13^ where long calls function as social signals, helping to space males with surrounding individuals. Among the three extant great ape genera (*Pongo*: orangutans, *Gorilla*: gorillas, *Pan*: chimpanzee and bonobos), only *Pongo* and *Pan* frequently produce structured loud vocal displays.^14^ In orangutans, long calls attract consorts and repel rivals,^15^ analogously to songs in other species.^16^ Conversely, in chimpanzees, “pant hoots” are primarily used for male cohesion^17^ and recruiting allies,^18^ while bonobos do not exhibit a homologous loud call type.^19^ Among great apes, orangutans are also the only ones currently known to organize long calls isochronously, that is, with call elements (“pulses”) regularly and equally spaced within a sequence.^20^ Orangutan long calls offer, thus, a unique opportunity to clarify whether non-song vocal displays can exhibit more complex rhythmic patterns, as found in humans and other singing primates.

Due to their loudness and conspicuousness, orangutan male long calls represent one of the best studied vocal behaviors in the genus, however, until now, research has strictly focused on long call pulses, known to be isochronous. Noted as early as 1922,^21^ males also often intersperse pulses with voiced inhales (“pants”). The role these elements may play in organizing long calls’ rhythmic structure remains wholly unstudied. Indeed, sound production through inhalation is common among non-human animals, more so than in humans.^22^ Famous examples include the braying of donkeys and the purring of cats. Inhaled sounds are also produced by various primates, including the impressive roars of howler monkeys,^23^ the songs of titi monkeys,^24^ and gibbons,^25^ and the pant-hoot vocalization of chimpanzees.^26^ Other inhale sounds also comprise the orangutan call repertoire.^27^^,^^28^ Nevertheless, ingressive phonation is also an important feature of vocal communication in humans, including non-verbal vocalizations,^22^ ingressive speech (presumed to have been historically more prevalent across cultures than today),^29^ and song, as used for example in traditional (e.g., Inuit Katajjaq, Japanese Rekkukara) and modern singing styles (e.g., beatboxing, “Inward Singing” by Tenacious D), including for the generation of double meter (e.g., “Earth Melodies” by Ekaterina Shelehova). The rhythmic study of both voiced inhales (“pants”) and exhales (“pulses”) in orangutan long calls is, thus, relevant for a comprehensive comparative approach to the evolution of song and music along the human lineage.

We hypothesize that if orangutan long calls exhibit a halving of the primary isochronous pulse, this evidence demonstrates that double meter in human song and music could have evolved from non-song vocal displays in ancestral hominids. Our findings reveal that orangutan calls not only display double meter produced through tempo variations, as found in singing primates, but also through controlled inhaled sounds, similarly to techniques in human musical traditions. This suggests that the ability to produce double meter is not unique to primate song but rather stems from ancestral vocal and respiratory mechanisms.

## Results

### Dual double meter

**Figure 1:**
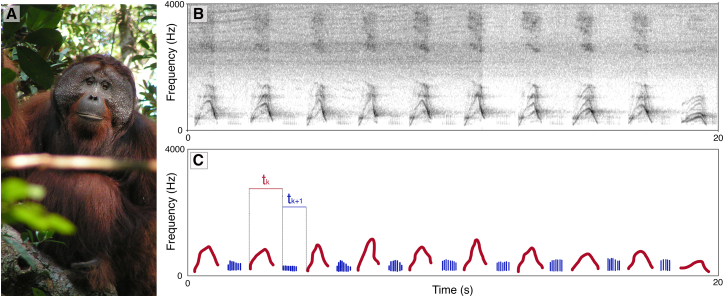
Orangutans’ long calls (A) A wild male orangutan (Photo by M. Hardus). (B) Raw spectrogram highlights a section of a long call containing a series of pulses and pants. (C) Schematic representation showing pulses (red) and pants (blue), and their t_ks_.

We investigated orangutan long calls (Figures 1A and 1B) according to four different levels of temporal organization, including pulses (voiced exhales) and pants (voiced inhales). We used GLMMs to analyze the rhythm and tempo of (i) pulses along a long call, (ii) a series of pulses and pants (Figure 1C), (iii) pulses interspersed by pants, and (iv) pants interspersed by pulses [the converse of (iii)].

**Figure 2:**
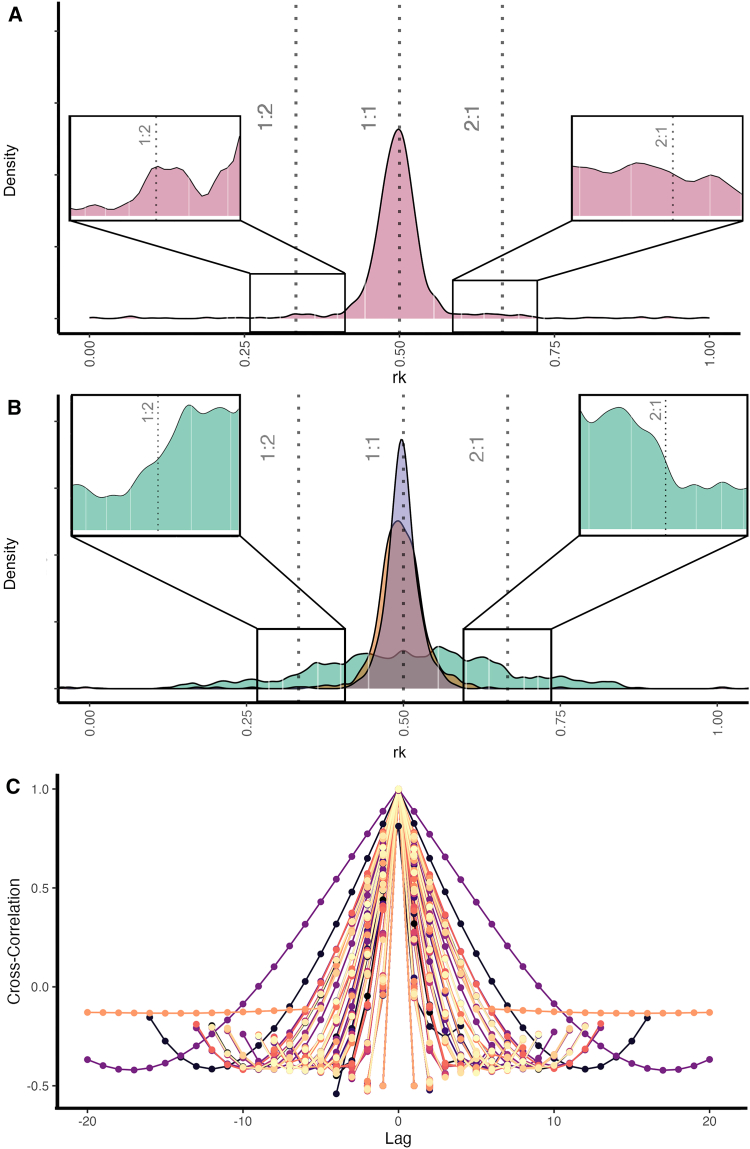
Rhythmic organization and cross-correlation in orangutans’ long calls (A) Main: density plot of r_k_ ratios overall (all calls). Insets: zoomed-in views of the highlighted regions corresponding to 1:2 and 2:1 ratios with adjusted kernel density parameters. (B) Density plot of r_k_ ratios for a series of pulse/pants (in green), pants only (in orange), pulse only (in purple). Insets: zoomed-in views of the highlighted regions corresponding to 1:2 and 2:1 ratios for pulse/pants series. (C) Values of cross-correlation between the timing of pulses and the timing of pants, at different time lags. Each line represents a different portion of the recordings where pulses are interspersed with pants. Dots along the lines represent time lags. Highest cross-correlation values are at lag = 0.
ii) Series of pulses and pants: Series matched three different rhythmic categories: isochrony (1:1off-1:1on, p < 0.0001), 1:2 ratio (1:2off-1:2on, p = 0.0005), and 2:1 ratio (2:1off-2:1on, p = 0.0301; Table 1). Voiced inhales divided pulse cycles halfway, doubling the sequence tempo and creating two subordinate rhythm categories at 1:2 and 2:1, hence, generating a double meter (Figure 2B). During periods of isochrony, double meter (1:2 and 2:1) occurred approximately one-third of the time. The density plot of the occurrence of rhythmic categories during the duration of long calls indicated that isochrony tended to occur more often at the beginning of long calls, while the 1:2 and 2:1 rhythmic patterns exhibited a bimodal distribution, peaking in the start and end section of long calls, following the occurrence rate of voiced inhales along a long call (Figure S1B. Occurrence of rhythmic categories during the vocal display]). That is, the production of inhales entailed the production of Pdouble meters.
iii) Pulses interspersed by pants: The timing between subsequent pulses contained in the pulse/inhales series was isochronous (1:1off – 1:1on, p < 0.0001). No other rhythmic ratios were present (Figure 2B).
iv) Pants interspersed by pulses: The timing of pulses extracted from a series of pulse/inhales was isochronous (1:1off – 1:1on, p < 0.0001; Figure 2B). The 1:2 rhythmic category was not significant (1:2off – 1:2on, p = 0.6200).

i) Pulses along a long call: First, we confirmed that long call pulses in our sample were indeed isochronous, as previously reported for a different population^19^ (1:1off-1:1on, p < 0.0001, Table 1). Notably, long call pulses also exhibited significant 1:2 and 2:1 ratios (1:2off-1:2on, p < 0.0001; 2:1off-2:1on, p < 0.0001; Table 1; Figure 2A). Double meter can, thus, be confirmed to be present in the non-song vocal displays of a nonhuman hominid. During periods of isochrony, double meter (1:2 and 2:1) occurred approximately 10% of the time. The density plot of the occurrence of rhythmic categories during long calls showed that isochrony tended to occur more often at the beginning of long calls, while 1:2 rhythmic patterns occurred more often midway and 2:1 rhythm toward the end (Figure S1A. Occurrence of rhythmic categories during the vocal display).

**Table 1:** Results of post-hoc comparison (*emmeans*) for the four GLMMs testing the presence of rhythmic categories

Rhythmic categories in long calls				
Factors	Estimate	SE	z.ratio	*p*
**(i) All Pulses**				

11off - 11on	−2.38	0.131	−18.209	**<0.0001**
12off - 12on	−1.31	0.232	−5.629	**<0.0001**
21off - 21on	−1.66	0.251	−6.597	**<0.0001**

**(ii) Series of pulses and pants**				

11off - 11on	−0.387	0.0841	−4.602	**<0.0001**
12off - 12on	−0.534	0.153	−3.481	**0.0005**
21off - 21on	−0.302	0.139	−2.169	**0.0301**

**(iii) Pulses [interspersed by pants]**				

11off - 11on	−3.2	0.255	−12.540	**<0.0001**

**(iv) Pants [interspersed by pulses]**				

11off - 11on	−2.42	0.179	−13.503	**<0.0001**
12off - 12on	0.573	1.15	0.496	0.6200

Bold denotes statistical significance (*p* < 0.05). i) null vs. full, χ2 = 967.9492, df = 7, *p* < 0.001. ii) full vs. null: χ2 = 591.6478, df = 7, *p* < 0.001. iii) full vs. null: χ2 = 326.1728, df = 3, *p* < 0.001. iv) full vs. null, χ2 = 396.4098, df = 5, *p* < 0.001.

### Pulse and voiced inhales tempo

Pulses interspersed by pants and pants interspersed by pulses occurred with the same tempo (pants vs. pulses, *p* = 0.9217; Table 2). The mean t_k_ between pulses was 2.02 ± 2.30 s (i.e., 0.5 Hz), while the mean t_k_ between pants was 1.92 ± 0.450 s (0.52 Hz). This was predicted because pants and pulses of the same series occurred in an alternating manner and were both isochronous. Long calls’ tempo doubled when pants alternated with pulses at 1.01 ± 1.62 s (0.99 Hz) (*p* < 0.001 for both cases; Table 2). This was consistent with the rhythmic analyses, where the presence of inhales between pulses generated a double meter.

**Table 2:** Post hoc comparison between the three levels of tempo: series of pulses and pants (“series”), pants extracted from series of pulses and pants (“pants”), and pulses extracted from series of pants (“pulses”)

Tempo of series of pulse/inhales					
Factors	Estimate	SE	df	t.ratio	p
Series vs. pants	−0.7385	0.0152	2383	−48.734	<0.001
Series vs. pulses	−0.745	0.0140	2375	−53.039	<0.001
Pants vs. pulses	−0.006	0.0173	2380	−0.385	0.921

Null vs. full, χ2 = 2332.983, df = 2, *p* < 0.001.

### Pulse and voiced inhales synchrony

Correlation analyses revealed that pulses and pants were produced in lockstep, where the highest value of cross-correlation for each recording portion was at lag = 0 (Figure 2C), which means that changes in the two series occur simultaneously and impact each other in real time. Cross correlation values ranged from 0.81 to 1 (Table S1. The highest values of cross-correlation for each pulse/pant series at relative lag, where 0 and 1 indicate no correlation and maximum correlation, respectively, demonstrating that the pulse and inhale time-series were highly synchronized.

Causality analyses revealed that 28% of the time, pulses predicted the tempo of pants (16 out of 57), and 19% of the time, pants predicted pulses (11 out of 57). No significant directionality was detected between pants and pulses in the remaining occasions (53%) (Table S2. Results of Granger causality test on pulse/pants series, related to Figure 2C), indicating that the rhythmic relationship between pulses and pants operated within a shared tempo, rather than one element predictably leading or following the other. The presence of bi-directional causality, as well as the absence of causality, indicated that the orangutan exerted dynamic airflow regulation during voiced production, which is in line with evidence for voluntary, real-time airflow and phonatory control in orangutans^30^^,^^31^^,^^32^^,^^33^ and other great apes.^34^

## Discussion

We confirmed that orangutan long calls are isochronous and exhibit more complex rhythmic patterns, namely, double meter (1:2 and 2:1 rhythmic ratios). This was achieved by two distinct mechanisms. First, through tempo variations in pulse production, analogously to singing a primate song.^7^^,^^10^^,^^11^ Second, by exploiting the natural inhale-exhale cycle of the phonatory-respiratory system, voicing is achieved both on the exhale and inhale. Voiced exhales (pulses) and voiced inhales (pants) took turns as rhythmic pacemakers and showed, on other occasions, a shared tempo, demonstrating real-time control of airflow and voicing. Causality and correlation analyses confirming high synchronization between pulses and pants, underpinned by enhanced airflow control and active voicing in real time, are key factors for the evolution of speech and song.^35^ Our findings demonstrate there was biological and behavior feedstock among ancestral ape-like hominids for the evolution of speech and song, supporting the view that these traits developed in humans gradually over time on the basis of shared ancestry and descent with modification. This is further corroborated by accumulating evidence showing that great ape vocal capacities have been largely underestimated^36^^,^^37^ and greatly surpass traditional assumptions.^38^^,^^39^^,^^40^^,^^41^^,^^42^^,^^43^^,^^44^^,^^45^

Our findings suggest a possible resolution to the long-standing question of how human song and music could have evolved from a non-singing ancestral species. Specifically, results show that rhythmic pulse in the form of a prototypical double meter may have been present in the vocal displays of non-singing hominids. This raises the possibility that double meter preceded the evolution of song in human ancestors, rather than being a consequence of it. In this perspective, complex rhythmic organization, often seen as a hallmark of musicality, could have originated from tempo arrangements in long-distance vocal displays, similar in structure to orangutan long calls, rather than from early singing-like behaviors.

Orangutans’ long calls are typically associated with high arousal levels and vigorous physical displays, such as snag crashing.^46^ In humans, it is proposed that ingressive sounds are difficult to suppress in states of high arousal, in part due to heavy breathing, making inhale sounds reliable markers of a vocalizer’s arousal state.^22^ It is possible that inhaling panting in orangutans provides a similar function (and putatively so also in chimpanzees).

Ingressive phonation is also far more common in humans than is generally presumed, being present in non-verbal (e.g., laughter, cry) and verbal communication (i.e., ingressive speech^29^^,^^47^), as well as in music (e.g., Inuit inhaled singing, Japanese Rekkukara, beatboxing). Ingressive phonation increases the perceived level of emotional intensity,^22^ and in speech, can work as a feedback marker during dialogue.^47^ Altogether, these convergent lines of evidence suggest an evolutionary connection between vocal mechanisms for the expression of emotion in hominids and the emergence of song and music in the human lineage. Within this process, ingressive phonation in particular, as an ancestral element of intense vocal displays, seems to have been co-opted into the first (proto)musical rituals. Our findings raise the intriguing possibility that rhythmic pulse in human song and music evolved from ancestral mechanisms of vocal control and breathing regulation engaged during high emotional displays.

### Conclusions

Findings show the human capacity to generate double meter in song, and by extension music, likely built on ancestral hominid non-song vocal behaviors and the phonatory-respiratory cycle for the production of rhythmically complex sequences. Findings inspire a new view on the role of ingressive phonation as a determinant mechanism organizing the form and function of animal vocal communication, a topic thus far largely overlooked but that will confidently contribute to an improved understanding of the evolution of rhythmic systems in nature, including the human evolutionary lineage.

### Limitations of the study

This study demonstrates the presence of double-meter rhythmic patterns in wild orangutan long calls. However, analyses were based on data from only three individuals recorded in natural conditions, which constrains the assessment of potential inter-individual variation. Collecting sufficient data from flanged males in the wild is inherently challenging due to their low density and wide-ranging behavior. An open question remains as to how consistent these rhythmic patterns are across individuals of the same species, and whether certain rhythmic categories are more commonly produced than others. Future studies comparing wild and captive individuals could help clarify these aspects and test the stability and variability of orangutan rhythm.

## Resource availability

### Lead contact

Chiara De Gregorio, chiara.de-gregorio@warwick.ac.uk.

### Materials availability

This study did not generate new unique reagents.

### Data and code availability

• Data: The datasets of annotated orangutans’ long calls temporal features associated with this publication is publicly available in a Zenodo data repository: https://doi.org/10.5281/zenodo.15350056. There are four different datasets: (1) tempo analyses, containing the duration of inhaled t_k_ and information on file and id. (2) Granger test analyses, containing the starting points of inhaled and exhaled parts of the long call, as well as information on file and id. (3) t_k_/r_k_ between pulses and pants, calculated between inhaled vocalizations (pants) and between exhaled vocalizations (pulses) extracted from a series of inhales and exhales. (4) r_k_ pulses overall, containing t_k_ and r_k_ values calculated overall (independently by the presence or not of inhales), together with info on id and file.
• Code: All associated code needed to reproduce the results in this publication has been deposited in a publicly accessible repository (https://10.5281/zenodo.15350056), which contains four scripts: (1) cross-correlation analyses, (2) Granger causality analyses, (3) R_k_ count analyses, and (4) T_k_ differences analyses.
• Additional information: Any additional information is available from the lead contact upon request.

## Acknowledgments

We thank the Indonesian Ministry of Research and Technology (RISTEK), the Directorate General of Forest Protection and Nature Conservation (PHKA), and the Gunung Palung National Park Bureau (BTNGP) for research permission in Indonesia. We thank the Tanjungpura University (UNTAN) for supporting the project and acting as an academic counterpart. We thank Cheryl D. Knott for research permission at the Cabang Panti research station. We thank Kim Nouwen, Eva Topelberg, and the staff of the Gunung Palung Orangutan Project for their assistance and hard work in the field. We thank Robert Essery for support during the indexing of audio recordings. We also thank Filippo Carugati for his support with visualization. ARL was supported by the UK Research & Innovation, Future Leaders Fellowship grant agreement number MR/T04229X/1.

## Author contributions

Conceptualization, C.D.G. and A.R.L.; data acquisition and curation: A.R.L.; methodology, C.D.G.; investigation, C.D.G.; writing, C.D.G. and A.R.L.; visualization, C.D.G.; funding acquisition, A.R.L.

## Declaration of interests

The authors declare no competing interest.

## STAR★Methods

### Key resources table

**Table:** 

REAGENT or RESOURCE	SOURCE	IDENTIFIER
**Deposited data**		

Code and Datasets	Zenodo	https://doi.org/10.5281/zenodo.15350056

**Experimental models: Organisms/strains**		

Bornean orangutan (*Pongo pygmaeus wurmbii*)	Gunung Palung National Park, West Kalimantan, Indonesia	RRID: NCBITaxon_9600

**Software and algorithms**		

R: a language enviroment for statistical computing v.4.3.1	R Core Team R: A Language and Environment for Statistical Computing R Foundation for Statistical Computing, 2023 Version 4.3.1	http://www.r-project.org/
Raven Pro 1.6.4	*Raven Pro: Interactive Sound Analysis Software.*The Cornell Lab of Ornithology, 2023 Version 1.6.4	https://ravensoundsoftware.com/software/raven-pro/
Adobe Photoshop	Adobe Photoshop, *Adobe Photoshop.*Adobe Inc., 2019 Version 23.0.0	https://www.adobe.com/products/photoshop.html
*glmmTMB* R package v1.1.9	Brooks et al.^52^	https://cran.r-project.org/web/packages/glmmTMB/index.html
*DHARMa* R package v0.4.6	Hartig, F. DHARMa: residual diagnostics for hierarchical (multi-level/mixed) regression models R package, 2020	https://cran.r-project.org/web/packages/DHARMa/index.html
*emmeans* R package v1.8.5	Lenth, R., emmeans: Estimated Marginal Means, aka Least-Squares Means, 2023 R package version 1.8.5	https://cran.r-project.org/web/packages/emmeans/index.html
*lmtest* R package v0.9.4	Zeileis and Hothorn^57^	https://cran.r-project.org/web/packages/lmtest/index.html

### Experimental model and study participant details

Three wild, adult flanged male orangutans (*Pongo pygmaeus wurmbii*, RRID:NCBITaxon_9600; Figure 1A), fully habituated to human presence, were observed and recorded non-invasively at the Cabang Panti Research Station (1°13’ S, 110°07’ E) in Gunung Palung National Park, West Kalimantan, Indonesia, between February and August 2010. Long calls are produced exclusively by adult males in this species. All research was conducted on free-ranging individuals under natural ecological conditions. We obtained all necessary research and data collection permits from the Indonesian Ministry of Research and Technology (RISTEK), the Directorate General of Forest Protection and Nature Conservation (PHKA), and the Gunung Palung National Park Bureau (BTNGP), and we received research permission from the Cabang Panti Research Station.

### Method details

Long calls (N = 47) were recorded using a Marantz Recorder PMD-660 with a Rode NTG2 Microphone between 7 and 20 m meters away from focal subjects. Orangutans are arboreal primates native to the equatorial and tropical rainforests of Southeast Asia and currently classified as Critically Endangered.^48^ They are predominantly solitary, loose, fission-fusion social organization compared to other great apes.^49^ Long calls (Figure 1B) play an important beacon function in these social settings, allowing coordination between dispersed individuals across mid and long distance, namely, mediating spacing with male rivals and potential female mates.^15^^,^^50^^,^^51^ Audio recordings were transferred to a computer with a sampling rate of 44.1 kHz. Spectrograms were visually inspected in Raven Pro (version 1.6; window type: Hann; 3 dB filter bandwidth: 124 Hz; grid frequency resolution: 2.69 Hz; grid time resolution: 256 samples). We annotated the starting point and duration of each long call pulse and interspersing pants (i.e., voiced inhales), when present.

### Quantification and statistical analyses

To characterise the rhythmicity of male orangutans’ long calls, we undertook four different level of investigation; We built time-series for rhythmic analyses considering (i) “all pulses” (i.e., all exhaled elements along a long call), (ii), “pulses and inhales” from pulse/inhale series (i.e., long call sections with alternating pulses and voiced inhales), (iii) “pulses only” from pulse/inhale series and (iv) “inhales only” from pulse/inhale series.

#### Tempo and r_k_ calculations

For all levels above, we calculated the duration of the intervals between the starting of an element and that of the following one (hereafter t_k_, following De Gregorio and colleagues,^10^; Figure 1C). For example, in the case of the “pulses and inhales”, the t_k_ was calculated from the beginning of a pulse to the beginning of the following voiced inhaled sound. We obtained 1243 t_k_ values for (i) all pulses, 1334 for pulses and inhales (ii), 561 for pulses only (iii) and 531 for inhales only (iv).

We then calculated the ratio of two subsequent t_k_ (hereafter r_k_), by dividing the duration of each t_k_ by the sum of its duration and that of the following one (following^3^). We obtained 1195 r_k_ values for all pulses (i), 1196 for pulse and inhale (ii), 422 for pulse only (iii) and 432 for inhales only (iv).

We divided the ratio distribution into on-integer and off-integer ratio ranges to investigate the occurrence of small integer ratios within levels. We centred the on-integer ratio range around 1:2 (or 0.33), 1:1 (or 0.50), 2:1 (or 0.66). Following previous works,^3^^,^^7^ a 1:1 on-integer ratio was considered when r_k_ falls between 0.444 and 0.555, and an off-integer ratio when r_k_ falls between 0.4 and 0.444, and between 0.5550 and 0.6. The range of a 1:2 on-integer ratio was considered between 0.308 and 0.364, and the off-integer ratio between 0.286 and 0.308, and between 0.364 and 0.4. For the 2:1 ratio, on-integer ratio was considered between 0.636 and 0.692 and an off-integer ratio between 0.6 and 0.636, and between 0.692 and 0.714. We then tallied all the ratios within the respective on-integer and off-integer ratio intervals for each long call.

#### Testing rhythmic categories

To test whether the rhythmic ratios (rk) fell more frequently within the on-integer than off-integer ratio ranges (i.e., whether they tended to concentrate around integer versus non-integer values), we used four Generalized Linear Mixed Models (glmmTMB package^52^), one for each level of analysis (i–iv), to assess whether the number of observations within predefined ratio boundaries exceeded chance levels based on a null distribution. This approach is conceptually equivalent to that of De Gregorio et al. (2021) but employs GLMMs to account for random factors in communicative behaviour. Moreover, this method is independent of visual peak detection and robust to overall distributional shape, capturing specific ratio patterns even in broad or skewed distributions. We checked the distribution of each response variable with the package fitdistrplus,^53^ and residual distribution and model dispersion with the Dharma package.^54^ GLMMs were fitted using a Poisson distribution (count variable) and we set ziformula = 1, to account for the presence of zeros in the datasets. We also included an offset variable weighting the r_k_ count based on the width of the bin in the probability density curve. We adopted a full versus null approach to test for the significance of our full models, comparing each model with the same one containing random factors only (Anova with chi-sq argument^55^). For all models, null and full significantly differed (Table 1), meaning that the fixed factors affected the distribution of the response variable, and therefore, we applied post hoc tests (emmeans^56^) to perform all pairwise comparisons for the levels of the fixed factors. For all models, we used as a response variable the r_k_ count and the r_k_ bin type as a fixed factor (factor levels: off1:1, on1:1, off1:2, on1:2, off2:1, on2:1). We used the file ID from which the ratios were extracted as a random factor.

#### Tempo comparison

We used a linear model to investigate differences in tempo between pulses and pants. We used as response variable the log-transformed duration of t_k_, and as fixed factor the type of t_k_ (i) series of pulses and pants (“series”), (ii) pants extracted from series of pulses and pants (“pants”), (iv) pulses extracted from series of pants (“pulses”). We used as random factor the identity of the portion of the recording from which the durations were extracted. This model was significantly different from its corresponding null model containing only the random factor (Table 1). We then applied a post-hoc test to perform all pairwise comparisons for all levels of the t_k_ type (emmeans package^56^).

#### Correlation and causality analyses

To investigate the relationship between the timing of the pulses and that of the pants, we ran a cross-correlation analyses using the function *ccf* function in R, with max lag = 20. This analysis identifies if temporal changes in the one series (i.e., pulses only) are related to changes in the other series (i.e., pants only), and if so, at what lag (or time shift) this relationship was strongest.

We also performed a Granger causality test (*GrangerTest* function in R^57^ − order = 1) on the timing of pulses only and pants only to assess whether a time series was effective in predicting the timing of the other one. We tested both directions, that is, if the timing of pulses only predicted the time of pants only (pulse ∼ pants, order = 1) and if the timing of pants only predicted that of pulses only (pants ∼ pulse, order = 1). We considered only the time series containing at least three observations (N = 52, 600s in total) and then counted the number of occurrences where p < 0.001, for both causality directions.
