# Supplementary material for: Twice times two: Dual mechanism for double rhythmic meter in orangutans and the evolution of human song
Source: iScience. 2025 Nov 28;29(1):114273. doi: 10.1016/j.isci.2025.114273 (PMC12765161; doi:10.1016/j.isci.2025.114273)
Supplement: Document S1. Figure S1 and Tables S1 and S2. [file mmc1.pdf]

iScience, Volume *29*

Supplemental information

Twice times two: Dual mechanism for double rhythmic meter in orangutans and the evolution of human song

Chiara De Gregorio and Adriano R. Lameira

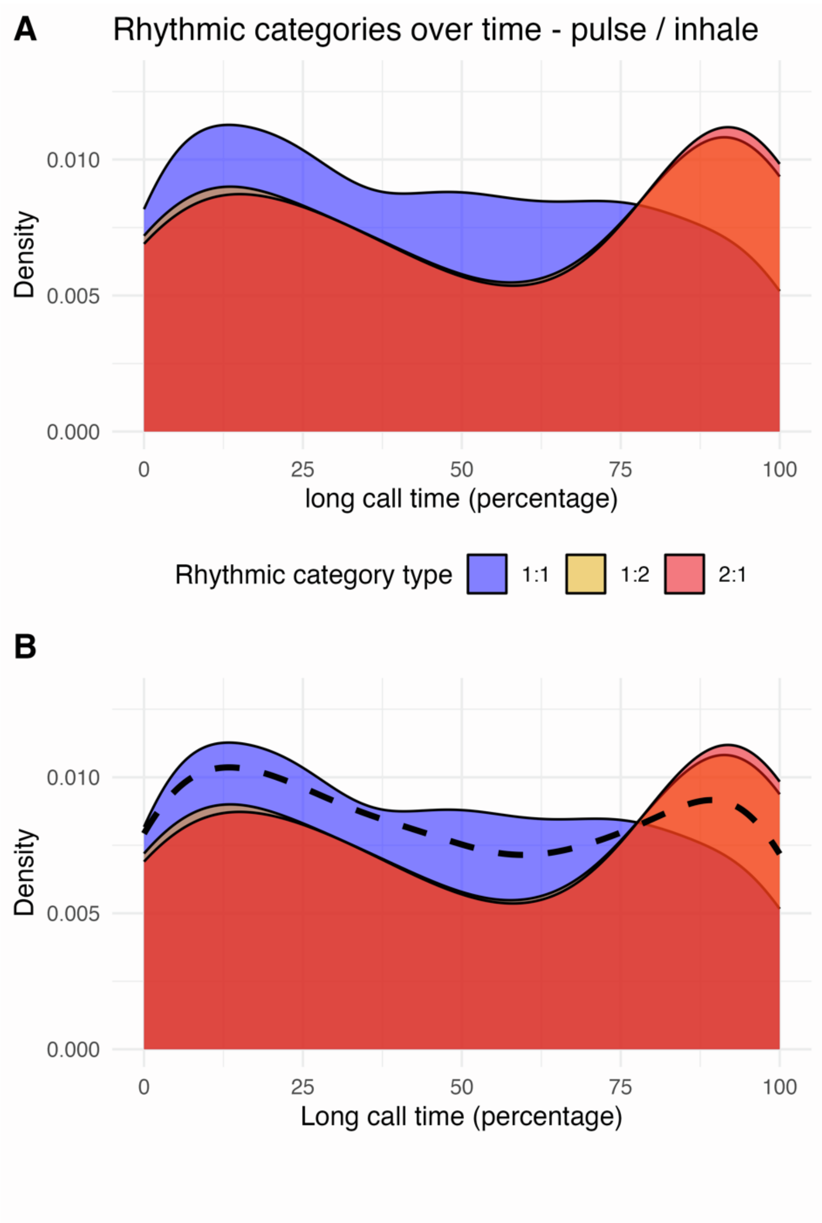

Figure SM1. Occurrence of rhythmic categories during the vocal display. (A) Density distribution of the occurrence of different rhythmic categories for all calls during the vocal display, time expressed as percentage. Blue = 1:1 ratio, yellow = 1:2 ratio, red = 2:1 ratio. **(B)** Density distribution of the occurrence of different rhythmic categories for series of pulse/pants during the vocal display. Dotted line represents the occurrence of pants during calling. Blue = 1:1 ratio, yellow = 1:2 ratio, red = 2:1 ratio.

chunk_inhale	lag	cross_corr
6	0	1
10	0	1
28	0	1
32	0	1
35	0	1

39	0	1
45	0	1
46	0	1
55	0	1
73	0	1
81	0	1
86	0	1
89	0	1
96	0	1
102	0	1
115	0	1
125	0	1
50	0	0,999999976
70	0	0,999998793
107	0	0,999997336
116	0	0,999997275
69	0	0,999997038
67	0	0,999993696
66	0	0,999981314
110	0	0,999954614
53	0	0,999953089
136	0	0,999948292
59	0	0,999948038
13	0	0,999946703
128	0	0,999923353
14	0	0,999919258
101	0	0,999917363
40	0	0,999906529
30	0	0,999906478
103	0	0,99990575
109	0	0,999897118
49	0	0,99989606
149	0	0,999893039
146	0	0,999889667
90	0	0,999887105
95	0	0,99986659
37	0	0,99986558
24	0	0,999847734
143	0	0,999836552
133	0	0,999834894
62	0	0,99982813
33	0	0,999821926

65	0	0,999805474
4	0	0,999791819
94	0	0,999784581
71	0	0,999772894
3	0	0,999768285
127	0	0,999755425
130	0	0,999720032
51	0	0,999706031
104	0	0,999697514
97	0	0,999677215
68	0	0,999674854
18	0	0,999674024
144	0	0,999648734
57	0	0,999638472
78	0	0,999616842
147	0	0,999611584
74	0	0,999592589
29	0	0,999584893
113	0	0,999538805
141	0	0,999504966
98	0	0,999500021
60	0	0,99946664
132	0	0,999440102
92	0	0,999413308
138	0	0,999377536
64	0	0,999338671
118	0	0,99932075
99	0	0,999294453
61	0	0,99927554
82	0	0,999256474
9	0	0,999226156
120	0	0,999159898
137	0	0,999120422
80	0	0,999100499
8	0	0,999066655
100	0	0,999023082
38	0	0,998942347
105	0	0,998874078
142	0	0,998857875
56	0	0,998853356
83	0	0,998446586
114	0	0,998264786

21	0	0,998188949
145	0	0,998029084
31	0	0,997014787
122	0	0,996765017
135	0	0,99618922
91	0	0,995174979
93	0	0,995009998
2	0	0,992141051
58	0	0,992094974
15	0	0,811895827

Table SM1. Highest values of cross correlation for each pulse/pant series at relative lag.

Note that for each chunk highest value is at lag = 0.

chunk_inhale	Res,Df	Df	F	Pr(>F)	type
2	2	-1	2,3915907	0,36542183	Pulse to Inhale
3	4	-1	4,78148881	0,11661846	Pulse to Inhale
4	10	-1	37,8174372	0,00016881	Pulse to Inhale
9	5	-1	0,93337919	0,38869596	Pulse to Inhale
13	2	-1	0,62505591	0,57411039	Pulse to Inhale
14	14	-1	8,61268387	0,01160727	Pulse to Inhale
15	2	-1	0,12430215	0,78421254	Pulse to Inhale
18	6	-1	3,59740032	0,11635781	Pulse to Inhale
21	2	-1	0,34929781	0,66018121	Pulse to Inhale
24	2	-1	0,01540456	0,92138788	Pulse to Inhale
29	4	-1	11,533763	0,04258202	Pulse to Inhale
33	5	-1	0,18338747	0,69052957	Pulse to Inhale
38	3	-1	3,74346738	0,19267213	Pulse to Inhale
40	7	-1	3,19227842	0,12422002	Pulse to Inhale
49	5	-1	0,62824653	0,47235847	Pulse to Inhale
51	4	-1	0,09150184	0,78203692	Pulse to Inhale
53	4	-1	494,399047	0,00019916	Pulse to Inhale
56	6	-1	2,51311717	0,17375837	Pulse to Inhale
58	4	-1	1,60438758	0,29468756	Pulse to Inhale
59	22	-1	7,0931308	0,0145443	Pulse to Inhale
60	4	-1	2,36016444	0,22205307	Pulse to Inhale
61	8	-1	20,3659447	0,00275556	Pulse to Inhale
62	11	-1	0,2429819	0,63270465	Pulse to Inhale
65	5	-1	3,36826802	0,14036418	Pulse to Inhale
66	2	-1	0,31354364	0,67503743	Pulse to Inhale

68	9	-1	24,7311069	0,00108895	Pulse to Inhale
71	6	-1	5,21658334	0,07118258	Pulse to Inhale
78	10	-1	0,01179763	0,91588943	Pulse to Inhale
83	3	-1	0,01962321	0,90142885	Pulse to Inhale
90	4	-1	0,2225638	0,66928427	Pulse to Inhale
94	4	-1	7,543218	0,07095066	Pulse to Inhale
97	6	-1	1,38187931	0,29270391	Pulse to Inhale
98	2	-1	0,0297145	0,89132813	Pulse to Inhale
99	2	-1	1,38120425	0,44882193	Pulse to Inhale
100	6	-1	67,4051197	0,00043642	Pulse to Inhale
101	11	-1	20,0813484	0,00117679	Pulse to Inhale
103	2	-1	0,87406843	0,52140557	Pulse to Inhale
104	4	-1	0,0184235	0,90062809	Pulse to Inhale
105	6	-1	8,21634729	0,03514157	Pulse to Inhale
110	7	-1	2,74153278	0,14885059	Pulse to Inhale
113	3	-1	12,0948193	0,07366103	Pulse to Inhale
116	22	-1	0,00477441	0,94556606	Pulse to Inhale
118	4	-1	0,23868781	0,65864064	Pulse to Inhale
120	5	-1	43,1936164	0,00277388	Pulse to Inhale
127	6	-1	3,38827576	0,12503066	Pulse to Inhale
128	9	-1	3,66174968	0,09202342	Pulse to Inhale
130	6	-1	33,8486615	0,00211812	Pulse to Inhale
132	4	-1	1,06365691	0,37824624	Pulse to Inhale
133	9	-1	5,55309733	0,04620992	Pulse to Inhale
136	9	-1	7,02045002	0,02927337	Pulse to Inhale
141	3	-1	0,44869512	0,57193639	Pulse to Inhale
142	3	-1	0,00707962	0,94060873	Pulse to Inhale
143	6	-1	1,13897824	0,3346645	Pulse to Inhale
144	10	-1	26,4804143	0,00060646	Pulse to Inhale
146	6	-1	0,29504388	0,61033559	Pulse to Inhale
147	4	-1	9,73590773	0,05246168	Pulse to Inhale
149	7	-1	0,10718129	0,75449734	Pulse to Inhale
2	2	-1	0,60858866	0,57823846	Inhale to pulse
3	4	-1	0,0195725	0,89760218	Inhale to pulse
4	10	-1	0,46316837	0,51327272	Inhale to pulse
9	5	-1	6,2670564	0,06652382	Inhale to pulse
13	2	-1	1528,8691	0,01627797	Inhale to pulse
14	14	-1	3,4392155	0,08648414	Inhale to pulse
15	2	-1	9,611275	0,19863985	Inhale to pulse
18	6	-1	1,47978034	0,27809538	Inhale to pulse
21	2	-1	47,0996547	0,09211399	Inhale to pulse
24	2	-1	4269,43334	0,00974229	Inhale to pulse

29	4	-1	10,9563382	0,04539094	Inhale to pulse
33	5	-1	0,13314196	0,73367005	Inhale to pulse
38	3	-1	0,53754825	0,53974176	Inhale to pulse
40	7	-1	0,06705709	0,8043259	Inhale to pulse
49	5	-1	0,14989138	0,71835588	Inhale to pulse
51	4	-1	9,3544157	0,05505806	Inhale to pulse
53	4	-1	0,06227573	0,81904594	Inhale to pulse
56	6	-1	0,58447492	0,47907072	Inhale to pulse
58	4	-1	21,1862386	0,01928016	Inhale to pulse
59	22	-1	1,64836618	0,21316704	Inhale to pulse
60	4	-1	1,31319879	0,33493928	Inhale to pulse
61	8	-1	0,49271087	0,50538251	Inhale to pulse
62	11	-1	1,56737657	0,23907882	Inhale to pulse
65	5	-1	0,98075872	0,3780715	Inhale to pulse
66	2	-1	807,71797	0,02239088	Inhale to pulse
68	9	-1	10,6182287	0,01155211	Inhale to pulse
71	6	-1	0,00972244	0,92528493	Inhale to pulse
78	10	-1	0,11346883	0,7439509	Inhale to pulse
83	3	-1	0,01688315	0,90850731	Inhale to pulse
90	4	-1	14,3686808	0,0322098	Inhale to pulse
94	4	-1	0,38239602	0,58010006	Inhale to pulse
97	6	-1	0,00980402	0,92497334	Inhale to pulse
98	2	-1	61,9730657	0,08043756	Inhale to pulse
99	2	-1	648,481489	0,02498666	Inhale to pulse
100	6	-1	8,89785824	0,03069622	Inhale to pulse
101	11	-1	0,25291504	0,62592044	Inhale to pulse
103	2	-1	1,3010688	0,45823301	Inhale to pulse
104	4	-1	3,47918709	0,1589988	Inhale to pulse
105	6	-1	6,34064196	0,05330053	Inhale to pulse
110	7	-1	15,4530741	0,00770353	Inhale to pulse
113	3	-1	0,00070457	0,9812341	Inhale to pulse
116	22	-1	0,00616674	0,93815106	Inhale to pulse
118	4	-1	3,77844047	0,14716033	Inhale to pulse
120	5	-1	0,02627081	0,87909871	Inhale to pulse
127	6	-1	4,16475771	0,09677332	Inhale to pulse
128	9	-1	0,18807009	0,67598471	Inhale to pulse
130	6	-1	0,35248486	0,57854837	Inhale to pulse
132	4	-1	0,20993369	0,67797085	Inhale to pulse
133	9	-1	3,6817954	0,09128425	Inhale to pulse
136	9	-1	0,0057859	0,94123515	Inhale to pulse
141	3	-1	1,14434233	0,39672814	Inhale to pulse
142	3	-1	11,0918119	0,07954753	Inhale to pulse

143	6	-1	6,21029654	0,05502303	Inhale to pulse
144	10	-1	0,12667991	0,73009979	Inhale to pulse
146	6	-1	10,323407	0,02364931	Inhale to pulse
147	4	-1	0,54608713	0,51348742	Inhale to pulse
149	7	-1	3,62184927	0,10571217	Inhale to pulse

Table SM2. Results of Granger causality test on pulse/pants series. Type = directionality of causality.
